# Novel mutations in the *FOXC1* gene in Japanese patients with Axenfeld-Rieger syndrome

**Published:** 2007-06-27

**Authors:** Nobuo Fuse, Kana Takahashi, Shunji Yokokura, Kohji Nishida

**Affiliations:** 1 Department of Ophthalmology and Visual Sciences, Tohoku University Graduate School of Medicine, Sendai, Miyagi, Japan; 2 Hiraka General Hospital, Yokote, Akita, Japan

## Abstract

**Purpose:**

Mutations in the forkhead transcription factor (*FOXC1*) gene have been shown to cause juvenile glaucoma associated with a variety of anterior-segment anomalies. The purpose of this study was to determine the ocular and genetic characteristics of two Japanese families with Axenfeld-Rieger syndrome (ARS).

**Methods:**

Genomic DNA was extracted from the leukocytes of six members of two families with ARS. The DNA from one exon of the *FOXC1* gene were amplified by polymerase chain reaction (PCR) and directly sequenced. The patients received standard systemic and ophthalmological examinations.

**Results:**

Sequence analysis of the *FOXC1* gene revealed a novel Ala85Pro missense mutation in Helix1 in family 1 and a deletion of 17 nucleotides (437-453) in Wing1 and Beta2 within the forkhead domain of the *FOXC1* gene in family 2. This deletion predicted a loss of the forkhead domain by a premature termination of translation. These mutations segregated with the ARS phenotype in an autosomal dominant pattern. The affected individuals in family 1 had posterior embryotoxon, iris hypoplasia, corectopia with early-onset severe glaucoma, atrial septal defect, aortic stenosis, and pulmonary stenosis. The affected members in family 2 had posterior embryotoxon and iris hypoplasia with early-onset glaucoma, and systemically they had hearing loss, hypertelorism, and telecanthus.

**Conclusions:**

A novel mutation in Helix1 and a novel deletion in Wing1 and Beta2 of the forkhead domain of the *FOXC1* gene have been identified in two families with ARS. *FOXC1* mutations cause a variety of developmental abnormalities in the anterior segment of the eye, and they also induce an elevation in intraocular pressures and early-onset glaucoma.

## Introduction

Dysgenesis of the anterior segment of the eye is a genetically heterogeneous developmental anomaly [1] resulting in a high risk of glaucoma. One such anomaly is Axenfeld-Rieger anomaly (ARA), which is occasionally associated with extraocular alterations such as maxillary hypoplasia, hearing loss, dental abnormalities, congenital heart defects, redundant periumbilical skin, and skeletal limb anomalies. ARA patients with these alterations are said to have "Axenfeld-Rieger syndrome" (ARS).

Nishimura et al. [2] identified a patient with primary congenital glaucoma who had chromosomal anomalies with a balanced translocation between 6p25 and 13q22. Cloning of the 6p25 breakpoint led to the identification of mutations in the *FOXC1* gene (formerly called *FKHL7*), and they demonstrated that mutations in the *FOXC1* gene were also detected in patients with Rieger's anomaly, Axenfeld's anomaly, and iris hypoplasia. These findings demonstrated that mutations in the *FOXC1* gene cause a wide variety of allelic disorders of the anterior segment such as iridogoniodysgenesis anomaly (IGDA) associated with glaucoma [3].

In a family with nine affected individuals in three generations, Mirzayans et al. [4] found that ARS was associated with a Gln23Stop amino acid substitution in the *FOXC1* gene. The affected individuals presented with different degrees of iris hypoplasia, displaced pupils (corectopia), and a prominent, anteriorly-displaced Schwalbe line (posterior embryotoxon). Peripheral iris strands were seen to bridge the iridocorneal angle.

Mutations in the *FOXC1* gene can cause a wide variety of phenotypes that share features with Axenfeld anomaly, Rieger anomaly, ARS, iridogoniodysgenesis anomaly, iridogoniodysgenesis syndrome, iris hypoplasia, iridogoniodysgenesis type 1, anterior segment mesenchymal dysgenesis, and congenital glaucoma [2-13]. Only a small number of studies have been reported on mutations in the *FOXC1* gene in the Japanese population [11-13]. The purpose of this study was to determine the ocular and genetic findings in two Japanese families with ARS.

## Methods

Genomic DNA was extracted from leukocytes of peripheral blood and purified by the Qiagen QIAamp Blood Kit (Qiagen, Valencia, CA). The purpose of the study and the procedures to be used were explained to all patients and an informed consent was obtained. The procedures used conformed to the tenets of the Declaration of Helsinki. This study was approved by the Tohoku University Institutional Review Board.

The proband in family 1 was a three-year-old girl who presented at three months of age with early onset severe glaucoma and who has systemic complications including atrial septal defect, aortic stenosis, and pulmonary stenosis. Her father also manifested early-onset glaucoma. The proband of the second family was a 38-year-old woman who was diagnosed with congenital glaucoma in both eyes at birth. Family 2 had five affected members and at least three affected members had similar features. Standard ophthalmic examinations were performed on all examined patients. Control subjects (52 men and 48 women; mean age 68.0±7.7 years) had IOP>21 mm Hg, normal optic discs, and no family history of glaucoma.

The forkhead domain (FHD), spanning amino acids 69-178, was amplified by polymerase chain reaction (PCR) using 0.5 μM concentration of a pair of primers in an amplification mixture (25 μl) containing 0.2 mM dNTPs and 0.5 U of ExTaq polymerase (Takara, Japan) with 30 ng template DNA. Oligonucleotide primers used for amplification of FHD of the *FOXC1* gene were forward (sense) primer 5'-AAC TCC CTG GGA GTG GTG CCC TA-3' and reverse (antisense) primer 5'-CGG CTC CTT GAG GTG CAG CCT-3'. The PCR products were purified using a PCR Purification Kit (PCR Kleen Spin Columns, Bio-Rad, Hercules, CA) after loading onto a 1.2% agarose gel. Purified fragments were directly sequenced by the BigDye^TM^ Terminator Cycle Sequencing Ready Reaction Kit (Perkin-Elmer, Foster City, CA) on an automated DNA sequencer (ABI PRISM^TM^ 3100 Genetic Analyzer, Perkin-Elmer).

## Results

### Family 1: Missense Mutation Ala85Pro

**Figure 1:**
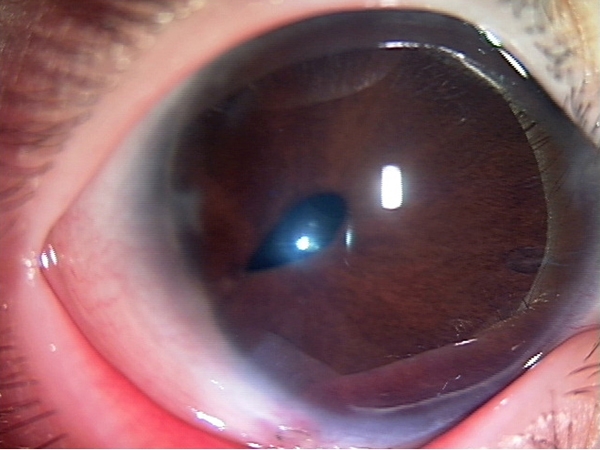
Right eye of proband 1. Right eye of proband 1 affected with ARS, displaying megalocornea, posterior embryotoxon, iris hypoplasia, and corectopia.

The proband in family 1 was a three-year-old girl who presented at three months of age with hazy megalocornea, posterior embryotoxon, iris hypoplasia, corectopia with early onset severe glaucoma, atrial septal defect, aortic stenosis, and pulmonary stenosis. The horizontal by vertical corneal diameters were 13.0 by 12.5 mm in the right eye (Figure 1) and 12.5 by 11.0 mm in the left eye. The patient underwent trabeculotomy three times in the right eye and two times in the left eye. Her father, who had posterior embryotoxon and iris hypoplasia with early-onset glaucoma, has been followed by a local ophthalmologist.

**Figure 2:**
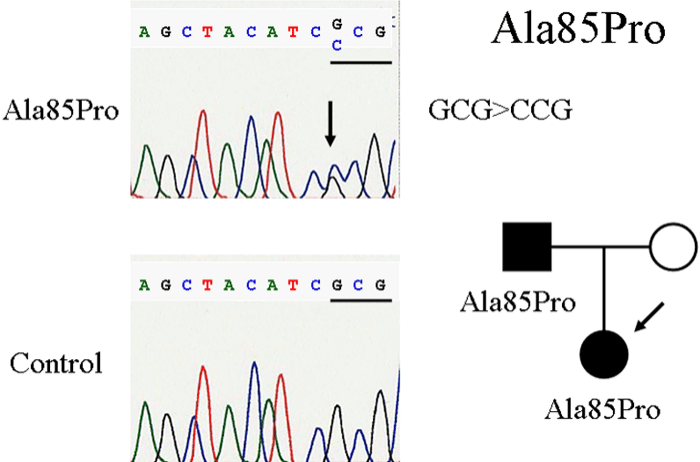
Comparison of mutated Ala85Pro with the normal gene sequence and family 1 pedigree. At top left, a fragment of the *FOXC1* sequence in an affected subject is shown. Ala85Pro is shown as a heterozygous novel mutation (G>C) at the first nucleotide in codon 85 which changed Alanine to Proline, seen at top right. At bottom left, a fragment of the *FOXC1* sequence in a normal subject is illustrated. The pedigree of family 1 is displayed at bottom right. The solid square indicates the father and the solid circle indicates the proband.

We screened the *FOXC1* sequence for mutations in this proband. After direct sequencing, a heterozygous novel point mutation (G>C) was found at the first nucleotide in codon 85 which changed alanine to proline (Ala85Pro; Figure 2). This novel missense mutation was identified in two members of this family, which is consistent with an autosomal dominant inheritance pattern. The Ala85Pro mutation was not found in 100 ethnically-matched control subjects.

### Family 2: Frame-shift mutation 437-453del17

**Figure 3:**
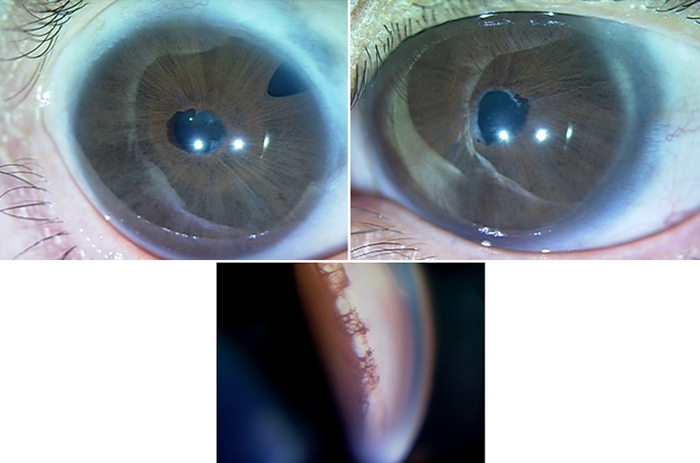
Clinical features of proband in Family 2. Top: The photo shows the eyes of proband affected with ARS. These eyes display megalocornea, posterior embryotoxon, iris hypoplasia, and right iridectomy (post-trabeculectomy). Bottom: Gonioscopic appearance of patients with proband illustrates iridocorneal angle anomaly. This appearance reveals tissue strands extending from peripheral iris to prominent Schwalbe's line and a high insertion of iris into trabecular meshwork.

**Figure 4:**
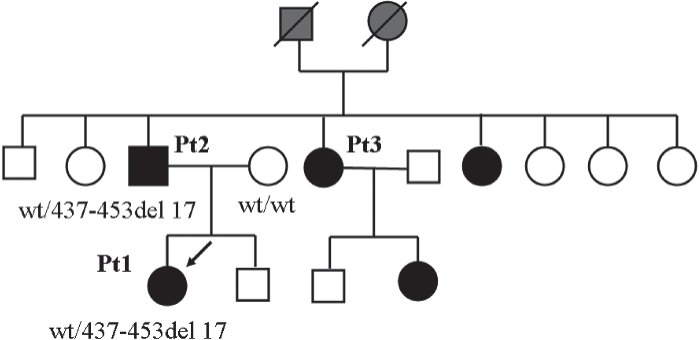
Pedigree of Family 2. Solid square indicates the father and solid circles indicate the proband and women. An arrow points to the proband. This family had five affected individuals.

**Figure 5:**
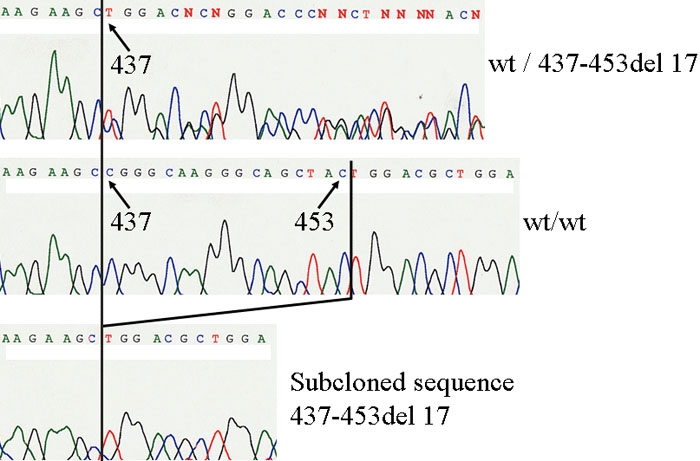
Deleted, normal, and subcloned gene sequence 437-453del17 in family 2. Top: A fragment of the *FOXC1* sequence in an affected subject is revealed to contain a wild sequence and deletions of 17 nucleotides from 437-453 bases (437-453del17). Middle: This graph shows a normal fragment of the *FOXC1* sequence. Bottom: A subcloned sequence shows the 437-453del17.

The proband of the second family was a 38-year-old woman who was diagnosed with congenital glaucoma in both eyes at birth. She underwent trabeculectomy once in the right eye and trabeculotomy/goniotomy three times in the left eye. The horizontal by vertical corneal diameters were 12.5 by 12.0 mm in the right eye and 14.5 by 12.0 mm in the left eye. She had posterior embryotoxon and iris hypoplasia with early-onset glaucoma (Figure 3). Family 2 had five affected members (Figure 4) consisting of patients 1, 2, and 3 as the proband, the father of the proband, and an aunt. All affected members (patients 1, 2, and 3) had similar features such as hearing loss, hypertelorism, and telecanthus. A heterozygous novel deletion, 437-453del17, was identified in the coding region of the *FOXC1* gene in two patients in this family (Figure 5). The mutation segregated with the disease phenotype and the distribution of the affected members was consistent with an autosomal dominant inheritance pattern. The 437-453del17 mutation was not found in 100 control subjects.

## Discussion

The *FOXC1* gene is a member of the forkhead/winged-helix family of transcription factors. These transcription factors contain a monomeric, 110 amino acid DNA binding domain, and forkhead domain (FHD). This motif was originally identified as a region of homology with the *Drosophila melanogaster* forkhead protein [14] and rat hepatocyte nuclear factor 3 protein (also known as *Foxa3*) [15]. The FHD is evolutionarily conserved and exists in a wide range of species from yeasts to humans [16]. This DNA-binding motif is a variant of the helix-turn-helix motif and consists of three helices and two large loops that form wing structures, Wing1 and Wing2 [2,6,17].

Different mutations in the *FOXC1* gene have been implicated in the pathogenesis of a wide spectrum of ocular disorders [2-13]. Mutant alleles segregate with the disease phenotype. Mutated residues are highly conserved across species implying that the mutations are probably pathogenic.

**Figure 6:**
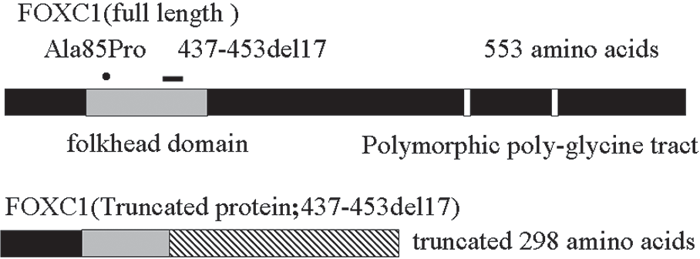
Summary of the mutations detected in this study. The hatched boxes indicate the location of the forkhead domain (FHD) within the *FOXC1* coding sequence. The two white boxes represent the locations of two polymorphic poly-glycine tract. The distribution of missense mutation Ala85Pro, and frame shift mutation (437-453del17) are illustrated. Ala85Pro and 437-453del17 existed in the FHD. The predicted protein translations are shown below the gene diagram for the frame shift mutations. The slashed boxes represent those areas of the protein that are translated differently than the normal FOXC1 protein; truncated 298 amino acids.

The mutations in our patients were found in the α-helix1 in the FHD, which is located at the amino acid position 85 in family 1 (Figure 6). Previous studies had noted that the NH_2_- and COOH-terminal boundaries of the FHD were critical for proper nuclear localization of *FOXA2* and *FOXF2* [18,19]. Two lesions are named the nuclear localization signal 1 (NLS1) and nuclear localization signal 2 (NLS2). The first region, NLS1, spans residues 77-93 where Ala85Pro is located. Saleem et al. [8] showed that Leu86Pro disrupts FOXC1 function severely revealing the importance of helix 1 in FOXC1 structure and function. Thus, there is a possibility that Ala85Pro reduces the DNA binding and significantly reduces transactivation.

In family 2, a deletion of 17 nucleotides (437-453) spans residues 146-151 and is located in Wing1 and Beta2 within the FHD of the *FOXC1* gene. This deletion generates a truncated 298 amino acids *FOXC1* protein instead of a 553 full length amino acid (Figure 6). Therefore, this deletion would disrupt the nuclear localization signal 2 (NLS2), which contains a basic stretch of amino acids at position ^169^RRRRRFKK^176^ at the COOH-terminal end of the FHD. Both NLSs are necessary for FOXC1 nuclear localization. The first region, NLS1, may serve as a nuclear localization signal (NLS) accessory domain and NLS2 could be the true nuclear localization signal [20]. Wing2 was suggested to play an important role in DNA binding and the transactivation capability of FOXC1 [9]. So, a deletion of 17 nucleotides (437-453) could possibly affect the DNA-protein interaction.

A haploinsufficiency of forkhead transcription factors has been shown to cause aberrant ocular development [2,21]. Considering these facts, we propose that these mutations may affect the migration and/or differentiation of the mesenchymal cells that contribute to the anterior segment of the eye [21] and the developmental processes including embryogenesis and tissue specific cell differentiation [16,22]. Only a small numbers of studies have reported mutations in *FOXC1* in the Asian population [11-13] thus, it is difficult to discuss extensive genotype-phenotype correlations. Kawase et al. identified four mutations: 26-47ins22, Ile91Ser, 286ins1, and Arg127His. The younger generations had iris hypoplasia with early-onset and severe glaucoma. The patient with frame-shift mutation, 26-47ins22, exhibited a more severe phenotype than patients with the other mutations [11]. Almost all of these mutations are located in the 110-amino-acid DNA binding domain and FHD, and are evolutionarily conserved and exist in a wide range of species from yeasts to humans [16]. All of the FOXC1 mutations could have the net of reducing FOXC1 transactivation [22]. In our case, frame-shift mutation, 437-453del 17, also showed high penetrance and slightly more aggressive glaucoma phenotype. Both frame-shift mutations, 26-47ins22 and 437-453del 17, make truncated proteins and haploinsufficiency may be correlated with the anterior-chamber defects of the eye.

Current medical therapies are not successful in decreasing the lower intraocular pressure or in preventing progression of glaucoma in patients with ARS. Only 18% of patients with glaucoma and either FOXC1 or PITX2 genetic defects responded to medical or surgical treatment [23]. The genotype-phenotype correlations of the *FOXC1* gene may help in establishing the prognosis of the disease processes and in understanding the mechanism associated with the various anterior segment dysgenesis caused by the *FOXC1* gene.
